# Formation and characterization of mechanochemically generated free lignin radicals from olive seeds

**DOI:** 10.3906/kim-2008-19

**Published:** 2021-04-28

**Authors:** Fatma DEMİR

**Affiliations:** 1 Department of Chemistry, Faculty of Science, Bilkent University, Ankara Turkey

**Keywords:** Lignin, olive seeds, mechanochemistry, mechanoradicals, radical scavenging activity

## Abstract

In this study, formation and quantification of mechanochemically generated free radicals of lignin were evaluated after the extraction of lignin from olive seeds and detailed lignin characterization was performed. Lignin was extracted from crushed olive seeds as an insoluble solid using Klason method. Isolated lignin was mechanochemically grinded under cryo conditions using Cryomill and particlesizes were determined by using Zeta Sizer, structural changes were followed by XRD and FTIR-ATR; thermal stabilities were tracked by TGA and DSC. In order to enable solubility demanding studies (such as ^1^H‑NMR and GPC), acylation of lignin was accomplished. ESR measurements were completed to prove the nature of the radicals. Free radicals cavenging activity of olive seed lignin was determined and quantified using 2-diphenyl-1-picrylhydrazyl (DPPH) method. Number of created mechanoradicals (per gram of olive seed lignin) was calculated from the corresponding UV‑Vis spectra. Finally, morphological changes of the lignin over cryomilling was evaluated using SEM.

## 1. Introduction

Olives are one of the most important terrestiral product which have been acknowledged as nobel and nutritious fruits all over the world and also as high potential agricultural crop for Mediterranean countries including Turkey. Over the last decade, the number of olive trees has increased by almost 60% and planted acreage has expanded by almost 25% in Turkey [1]. In connection with this, olive oil production and the biomass generated by the olive cultivation are also expanding. Although valuable industrial by-products could be extracted out of the olive production waste, it is basically burned out for energy production [2]. The mass of the solid waste, generated from olive extraction, is mainly olive seeds (seed husks) (18%–22% of the olive weight) [1] which is predominantly lignocellulosic material with hemicellulose (26.8%), cellulose (36.4%) and lignin (26% of dry weight) [3]. Among them, lignin -the main interest of this work- is the most abundant phenolic organic material on earth and exists in the cell wall in vascular and support tissues of the plants, bearing mechanical strength and tension. Lignin is an underutilized industrial raw material, although it is as an abundant renewable source and a by-product of many industries.

Due to its hindered phenolic hydroxyl groups, lignin is known as an antioxidant material and acts as a radical scavenger which stabilize, induce, retard or prevent oxidation process induced by oxidizing species such as free radicals [4–7]. In this sense, antioxidant compound extraction and characterization technologies from natural sources, including lignin, are being extensively investigated with the aim of using them as natural additives for functional materials in drug, food, packaging and polymeric industries [8]. 

**Figure 1:**
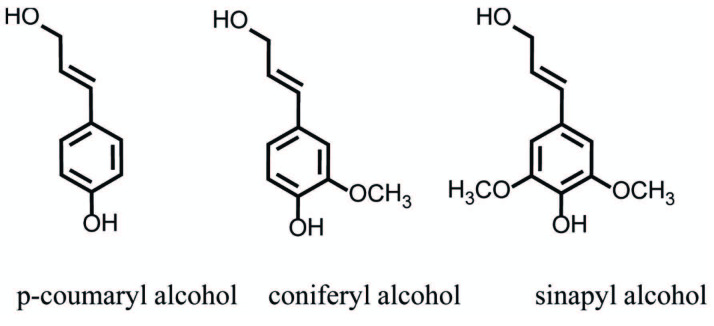
Chemical structures of coumaryl, coniferyl and sinapyl alcohol units, as building blocks of lignin.

Chemistry of lignin is complex, since it is a randomly crosslinked and amorphous biopolymer with high polyphenolic content. Despite the lack of a defined chemical structure; it contains mainly three common precursors; p‑coumaryl alcohol, coniferyl alcohol and sinapyl alcohol (Figure 1). Lignin has plenty β-O-4 linkage (a bond between β- carbon of one monolignol unit and phenolic hydroxyl of the other) in addition to other types of ether bonds such as α-O-4, 4-O-5 and C-C bonds β-β, 5-5, β-5 and β-1 and etc.

Lignins vary in structure depending on their extraction methods and the type of source (hardwood, softwood or annual crops) [9–11]. It is well known that purified ‘milled wood lignin’ can be extracted by ball milling (in the presence of neutral solvents) [12]. Bjorkman claims that it is not evident whether milling “opens up” the wood structure to allow lignin to diffuse or if lignin is set free by breaking covalent bond [12]. For the isolation of lignin from pulp, several different enzymatic, chemical and mechanical methods have been developed and applied [13]. Among others, Klason method is the most robust and well-known isolation method by which cellulose and hemicellulose are removed by 72% sulfuric acid at high temperature and pressure [14].

Regarding the antioxidant property [15,16] and the total peroxy radical-trapping ability [17], most of the research have been done for olive and olive oils, but not much of attention have been paid towards olive seeds which are low-cost waste of olive oil industry. 

Solely, Alu’datt et al. conducted a study to evaluate the antioxidant activities of phenolic compounds extracted from olive seeds [18]. They found a positive correlation between the phenolic compounds and the antioxidant activities of olive seeds. Moreover, the predominant phenolic compounds in olive seeds were reported to be present in free form as compared to only a small percentage of bound phenolic compounds [18]. Although their study focus on the optimization of the phenolic compound extraction and characterization, without associating the antioxidant activity with lignin content, the outcomes of the study led toward the investigation of free radical scavenging ability of olive seed lignin and the possibility to enhance such an ability by mechanochemical output. Mechanochemistry enables solvent free, green and more sustainable solutions as compared to conventional solvent-dependent chemistry methods. 

As summarized in one of the first mechanochemistry publication [19], purely mechanical cutting or breaking action, such as grinding, can cleave covalent bonds, and the free radicals are formed. It has been explained that the formation of free radicals by mechanical breaking, cutting, grinding, scratching or polishing result chiefly through a homolytic breaking of covalent bonds and formation of ions or radical-ions from free radicals [19]. Later, Sakaguchi et al. reported that mechanoradicals produced by homolytic scission of carbon-carbon bonds in the main chain of the polymer and proved it by the ESR studies [20]. Cleavage of β-O-4-linkages in lignin via ball milling which provides fragments of lower molecular weight lignin [21] and the formation of mechanoradicals of cellulose resulting from the breakage of covalent bonds during mechanochemical treatment were also proved by literature [22].

**Figure 2:**
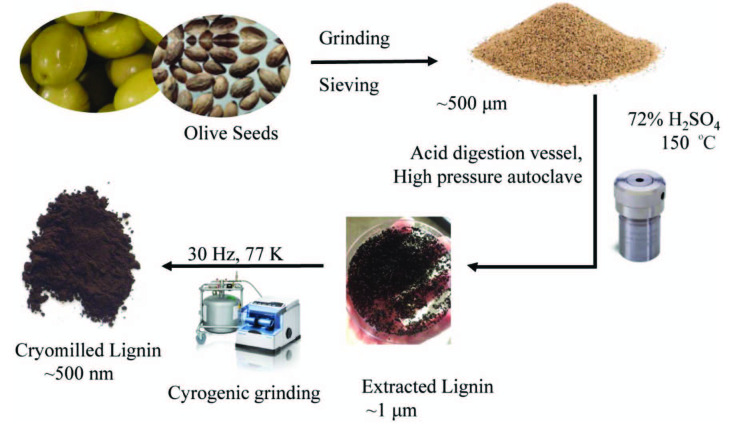
Illustration of lignin isolation from olive seeds and milling in cryo conditions (cryomilling).

Within the scope of this work, it is aimed to follow a clear method to isolate lignin from olive seed powder and a detailed characterization of extracted lignin (Figure 2). Cryomilling, an advantageous mechanochemical technique is used to increase the number of phenolic ends, thus the number of radicals which are already present in olive seed lignin. By applying mechanochemical external force, intramolecular covalent bonds of lignin are broken and reactive ends by homolytic cleavage are formed [23]. Milling in cryo conditions (–196 °C) both prevents samples from thermal change and also increases the lifetimes of the produced mechanoradicals [24]. ESR proves the mechanochemically created radicals to have free radical nature. Generated mechanoradicals are then trapped by radical scavenger 2-diphenyl-1-picrylhydrazyl (DPPH). The amount of the trapped DPPH is correlated with the amount of the generated mechanoradicals from olive seeds.

## 2. Results and discussions

### 2.1. Structural and morphological studies of lignin samples

**Figure 3:**
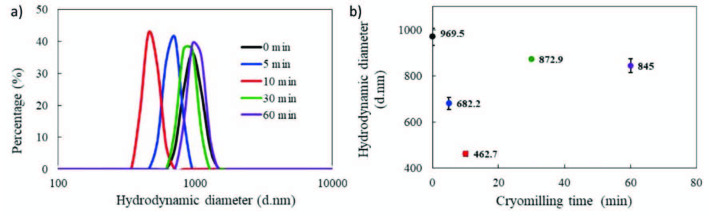
a) Particle size distribution, b) hydrodynamic diameter of lignin samples extracted from olive seed before (0 min) and after cryomilling for 5 to 60 min.

The hydrodynamic diameter (size distribution) profiles of lignin samples with different milling times are presented in Figures 3a and 3b. It is worth to note that the hydrodynamic diameter was the apparent size and not the actual size of the particles. But it could be used as an important reference value of size distribution of the samples, as it has been used in many studies [25,26]. Figures 3a and 3b indicate that as the cryomilling time increased, a reduction of the hydrodynamic diameter of lignin samples were observed (969–682 and 462 nm for 0, 5 and 10 min, respectively). Thirty and sixty minutes of cryomilling does not seem to further decrease the particle size, probably due to aggregation of the particles.

**Figure 4:**
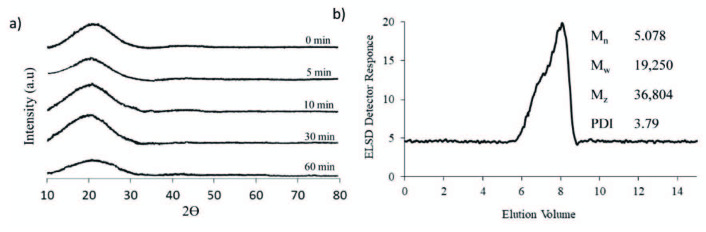
a) X-Ray diffraction patterns of olive seed lignin samples, and b) GPC chromatogram of acylated lignin (in order to ensure sufficient solubility, lignin samples are first cryomilled and then acylated prior to GPC).

The results obtained from X-ray diffraction analysis (XRD), performed to olive seed lignin samples before and after cryomilling, are displayed in Figure 4a in the form of normalized diffractograms. The diffractograms of lignin samples (noncryomilled and cryomilled with increasing times) allow to observe an amorphous behaviour without patterns of crystallinity. The diffractograms exhibited a wide band, with more well‑defined peaks in the region of 10 ≤ 2Ɵ ≤ 30. The maximum diffraction angle was determined to be around 20°, in well agreement with literature [27]. 

In order to increase the solubility of the lignin and enable further characterization (such as GPC and ^1^H-NMR), derivatization is typically accomplished by acylation with slight modification from literature [23,28]. Cryomilled olive seed lignin was acylated in the presence of trimethylacetylchloride and acylated lignin was recovered as solid powder (Scheme 1, see experimental section for details). 

**Scheme:**
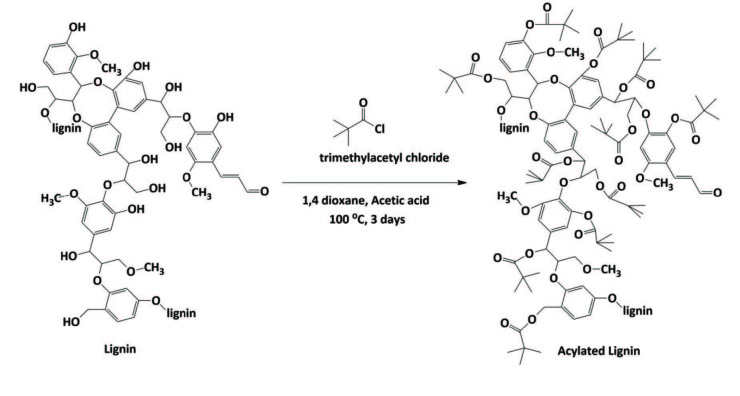
Proposed acylation of lignin by trimethyacetyl chloride reagent.

The molecular weight of the acylated lignin were estimated using GPC (Figure 4b). The number average molecular weight (M_n_), weight average molecular weight (M_w_) and z‑average molecular weight (M_z_) were estimated to be around 5078; 19,250 and 36,804 respectively, and polydispersity index (PDI) was calculated as 3.79. 

**Figure 5:**
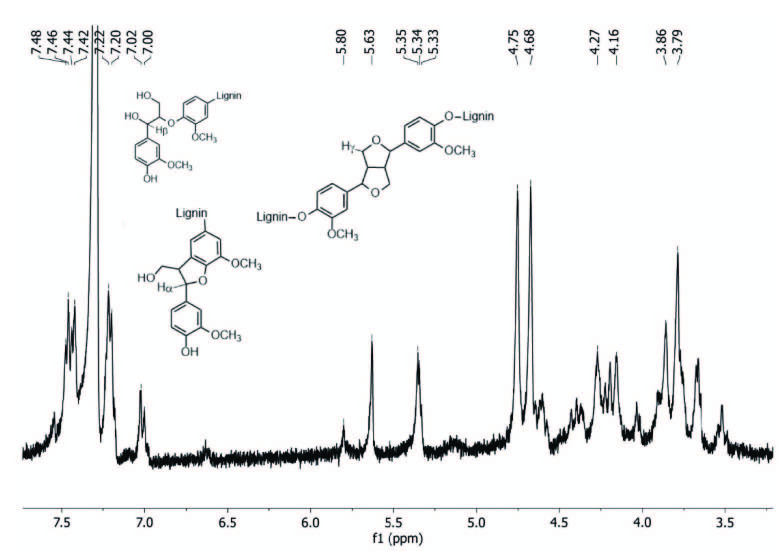
^1^H-NMR spectrum of acylated cryomilled olive seed lignin in CDCl_3_. Inset structures show the H_α_, H_β_ and H_γ_ protons in lignin molecule (for chemical shifts of peaks and their assignments see Table 1).

Figure 5 shows the ^1^H-NMR spectrum of acylated olive seed lignin (cryomilled for 60 min) in CDCl_3_. Table 1 summarizes the ^1^H-NMR assignments of lignin after acylation reaction by trimethylacetyl chloride reagent (as shown in Scheme 1) and the conclusions are in line with literature reports [13,29].

**Table 1:** Assignment of peaks in the ^1^H-NMR spectrum of acylate

δ (ppm)	Assignments
7.42–7.47	Aromatic protons in benzaldehyde units and aromatic protons located ortho tocarbonyl groups
7.20–7.22 and 7.00–7.02	Aromatic protons in guaiacyl units
5.63, 5.80	Noncylic benzylic region
5.33–5.35	Hα in β-5 structures and noncyclic benzyl aryl ethers
4.75	Hγ in β-β structures (methylnene protons in cinnamyl alcohol units)
4.68	Hβ in β-O-4 structures (methylene protons in cinnamyl alcohol units)
4.16–4.27	Hγ in several structures
3.86–3.66	Protons in methoxyl groups

**Figure 6:**
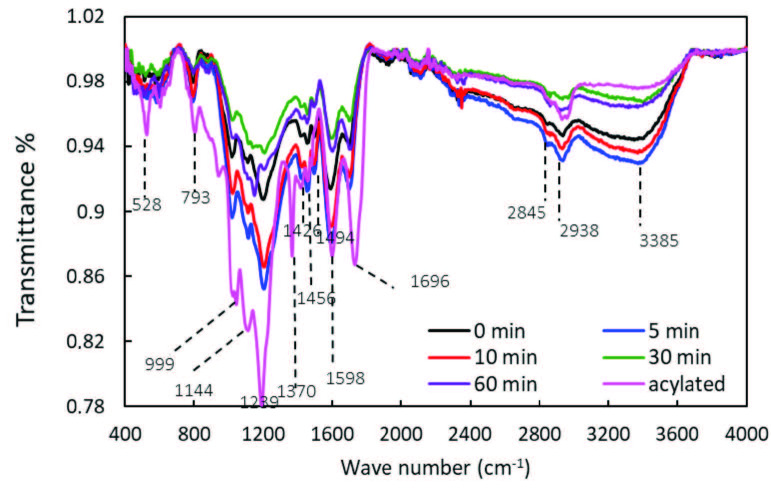
Normalized FTIR-ATR spectra after baseline correction of lignin samples. before (0 min) and after cryomilling for 5 to 60 min and after acylation.

The FTIR-ATR of extracted lignin (noncryomilled and cryomilled with different times) samples clearly indicate the presence of various characteristic functional groups of lignin as it was also confirmed by the literature [30,31]. In the FTIR-ATR spectra, as shown in Figure 6, a broad peak at 3385 cm^-1^ and a sharp peak at 2938 cm^-1^ were attributed to -OH and methyl/methylene groups, respectively. A symmetric stretch for CH3 of methoxyl group appeared at 2845 cm^-1^. A small peak at 1696 cm^-1^ was assigned to carbonyl stretching of unconjugated ketones and carbonyl groups. Sharp peaks at 1598 cm‑1, 1494 cm^-1^, 1456 cm^-1^, and 1426 cm^-1^ represents aromatic skeletal vibrations. Peaks at 1209 cm^-1^ and 1111 cm^-1^ were attributed to syringyl ring breathing with C=O stretching and C-O stretching for secondary alcohols, respectively. Aromatic C-H in place deformation was observed at 1026 cm^-1^ while aromatic C-H out of plane deformation was observed at 793 cm^-1^. Although the intensity of these characteristic peaks was changing with different cryomilling times, no changes in FTIR-ATR spectra were observed; thus, it was confirmed that no structural change was caused upon cryomilling. After the cryomilling, a clear increase on the intensities of some characteristic peaks which were attributed to the formation of mechanoradicals due to bond breakages, were observed. For example, more –OH groups were observed (3385 cm^-1^) for 5 and 10 min of cryomilling than the noncryomilled lignin. Acylation caused no obvious difference in the main absorption peaks (the peaks at 1598 cm^-1^, 1456 cm^-1^, and 1426 cm‑1, corresponding to aromatic skeleton vibrations); this indicated that the main structure of lignin did not change during esterification process. The success of lignin esterification was proved by the appearance of a new peak at 1370 cm^-1^ (due to the introduction of acyl groups), the intensity decrease of 3385 cm^-1^, transfer and intensity increase of 1696 cm^-1^ to 1735 cm‑1 (aliphatic ester links), appearance of a small shoulder at 1762 cm^-1^ (phenolic ester links), intensity increase of 1209 cm^-1^, 1111 cm^-1^, and 1026 cm^-1^. 

**Figure 7:**
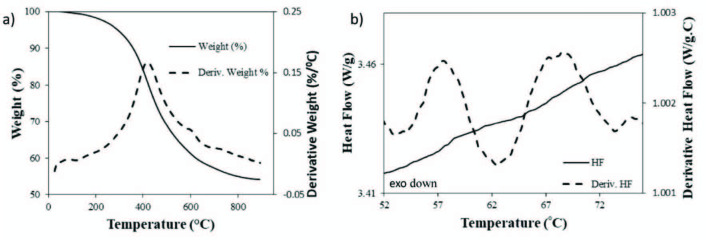
a) TGA, and b) DSC thermograms of lignin samples extracted from olive seed (heating rate: 20 °C/min).

As depicted in Figure 7a), the weight of the olive seed lignin was recorded while the sample was heated with a heating rate of 20 °C/min by TGA. About 45% of the mass was lost during the heating of the sample up to 800 °C and mass loss started before 200 °C and become fastest around 415 °C as indicated by the derivative weight signal.

Glass transition temperatures are crucial transitions for the evaluation of the amorphous region of the sample and it was followed as stepwise increase of the heat flow signals and also can be easily determined by the derivative heat flow signal as peaks. As shown in figure 7b), two different glass transition temperatures (T_g_) were observed during the first heating of the olive seed lignin, first at 57.5 °C and the second at 68.5 °C. These two different T_g_ were attributed to two different amorphous regions in the bulk lignin samples.

**Figure 8:**
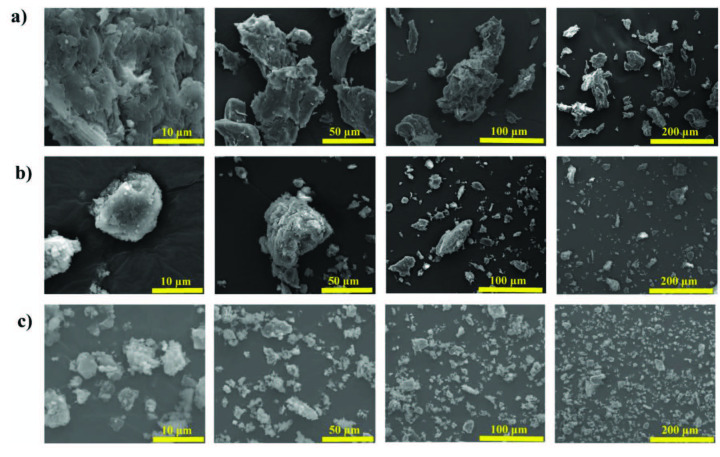
SEM images showing the morphology of a) olive seed powder as received, b) lignin extracted from olive seed, and c) 60 min cryomilled olive seed lignin samples at several magnifications. The images show the reduction of the particle size of the olive seed lignin after cryomilling.

Figure 8 compares the morphological changes of the olive seed powder as received and extracted lignin samples before and after cryomilling. SEM images show that the olive seed powder is about 50–150 μm size with external surface containing many cracks, cavities and layers. The surface of the bulk lignin samples also seems to have fractures and cracks on layered nature and the sizes are estimated to be around 10–50 μm. As expected, cryomilling decreased the size of the bulk lignin samples (down to 1–20 μm), and no damage or deformation were detected on the morphology of olive seed lignin. 

### 2.2. Identification of the generated mechanoradicals via electron spin resonance (ESR) spectroscopy 

**Figure 9:**
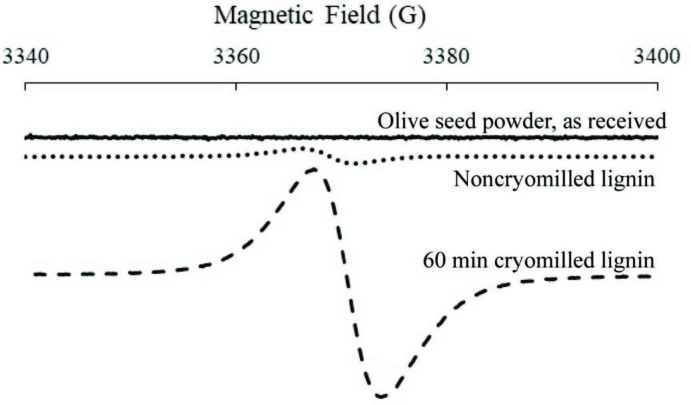
ESR spectra of olive seed powder as received, noncryomilled and 60 min cryomilled lignin extracted from olive seed powder.

In general, a free electron shows a g-value of g_e_= 2.003 ± 0.002 [32], and if the electron is located in a molecule, the g value shifts away from g_e_ depending on the structure of the molecule. ESR spectrum of olive seed powder as received, 60 min cryomilled and noncryomilled lignin extracted from olive seed are compared in Figure 9. Noncryomilled and 60 min cryomilled olive seed lignin samples showed a singlet signal at a g-value of 2.0047 and 2.0025, respectively, while as received olive seed powder show no signal, most probably due to very low number of radicals in the biomass. The g-value of the noncryomilled lignin is in good agreement with the literature and reported to be a semiquinone type radical [32–34]. Cryomilled sample represents much higher intensities in the spectra due to increased number of radicals generated during cryomilling. The g-value of the cryomilled sample shifts from 2.0047 to 2.0025 which indicates the formation of new mechanoradicals probably due to bond breakage on the main chain as reported for hydrocarbons [35–37]. The observed singlet spectra at the g‑value of 2.0025 were assigned to phenoxy radicals, which were generated by homolytic scission of the alkyl phenyl ether, alkyl-O-phenyl bonds connecting the three-dimensional networks of the lignin molecules [31,34]. Among the competing bond breakage possibilities (α and β-alkyl, aryl, ether, C-C, C-O, C-OCH_3_) along the main chain, the most dominant option is thought to be the β-O-4 bond breakage which are also supported by the FTIR-ATR results. 

### 2.3. Evaluation of free radical scavenging activity and antioxidant activity (AA%) via the DPPH method 

**Figure 10:**
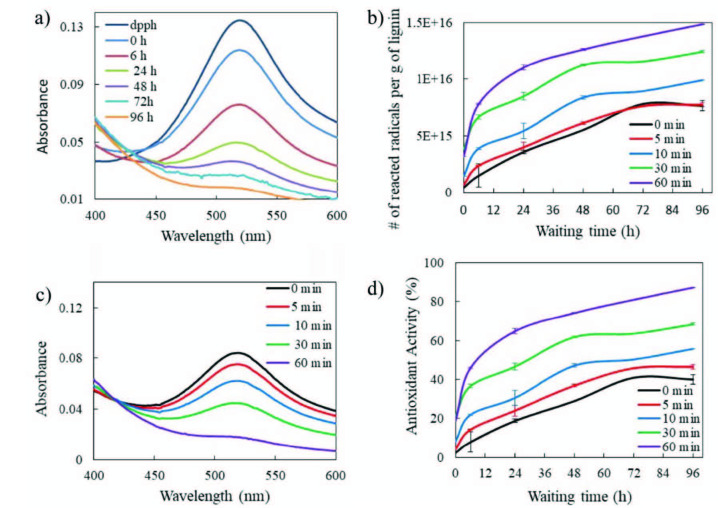
a) UV-Vis spectra of the DPPH solutions without and with lignin (cryomilled for 60 min) after waiting time up to 96 h, b) number of reacted radicals per gram of lignin for each cryomilling time calculated from the corresponding spectra, c) the UV‑Vis spectra of the DPPH solutions after 96 h, when almost all lignin mechanoradicals are scavanged by DPPH molecules, and d) antioxidant activity (%) of the lignin samples cryomilled different times over a waiting time up to 96 h.

The potential for free radical scavenging activity of olive seed lignin, using the DPPH method, was evaluated by a method described by Blois [37] and then Brand-Williams et al. [38]. Mainly, antioxidant activities of lignin samples were determined using the free radical, DPPH. In its radical form, DPPH has maximum absorption band at 519 nm, but upon reduction by an antioxidant (in this case olive seed lignin), the absorption intensity decreases and the consumption of DPPH radicals can be calculated with the amount of antioxidant depending on the reduction of UV absorption band. In order to ensure that only mechanoradically created radicals consume DPPH in the solution, noncryomilled sample was also exposed to DPPH solution as a control solution. Both noncryomilled and cryomilled (for 5, 10, 30, and 60 min) samples were exposed to DPPH for an indicated waiting time (from 0 to 96 h) and enough time is given for the reaction between the lignin mechanoradicals and DPPH for the adequate diffusion and interaction. Over time, DPPH is consumed by the mechanoradicals and the amount of consumption is followed by the decrease in the absorbance of the DPPH (Figure 10a), enabling the calculation of the reacted number of radicals per gram of lignin against waiting time (Figure 10b). It is worth to note that the control solutions which contains only DPPH (without any lignin) in ACN was also measured at the same concentrations and no bleaching were detected under the same conditions. As shown in Figure 10b, the amount of radicals increase with increasing waiting time (faster at the start, then slowly) but even after 2 days, the diffusion of the radicals and the interaction between the mechanoradicals and DPPH still continues. It also represents that for longer cryomilling times, more free OH, due to mechanoradical generation, are formed due to bond breakages of the lignin. It is worth to note that even the noncryomilled olive seed lignin has some radical scavenging activity from the beginning (Figure 10b zero min), which confirms the antioxidant nature of the lignin (in good agreement with literature [18]). The scavenging activity of the noncryomilled sample has enhanced over waiting time in DPPH solution, due to the migration of the present radicals from the bulk to the surface. Ninety-six hours of waiting time seems to be long enough for the radical diffusion; thus, it is selected as a comparison time where a plateau is reached for each cryomilling time as shown by the trends in Figure 10b (no further significant consumption is expected afterwards). Figure 10c shows the UV-Vis spectra of the lignin samples after 96 h of exposure to DPPH solutions, noncryomilled sample (0 min) has the highest intensity of DPPH, since the least amount of lignin mechanoradicals were present, least amount of DPPH is consumed and thus, the highest amount of DPPH is detected by UV. Accordingly; 60 min of cryomilling creates the highest number of mechanoradicals which consumed the greatest number of DPPH molecules in the solution that resulted in the lowest intensity in UV-Vis. 

Table 2 summarizes the amount of mechanochemically created radicals by cryomilling. As shown, the amount of radicals per gram of noncryomilled lignin is calculated to be around 7.64 × 10^15^ while it is around 1.48 × 10^16^ for 60 min cryomilled lignin. By the subtraction, 7.16 × 10^15^ is calculated to be the number of mechanochemically generated radicals at the end of 60 min of cyromilling. It can be concluded that around 1.94 times more radicals are created with cryomilling for 60 min compared to noncryomilled sample after 96 h of waiting in DPPH solution. 

**Table 2:** Number of reacted radicals per gram of olive seed lignin created during cryomilling of 5, 10, 30 and 60 min after 96 h of waiting in the DPPH solution and the ratio of them with the noncryomilled lignin sample.

Cryomilling time(min)	Calculated number of mechanoradicalsper gram of lignin	Ratio
0	7.64 × 1015 ± 4.54 × 1014	-
5	7.74 × 1015 ± 1.95 × 1014	1.01
10	9.91 × 1015 ± 1.86 × 1013	1.30
30	1.24 × 1016 ± 9.58 × 1013	1.62
60	1.48 ×1016 ± 1.97 × 1013	1.94

Additionally, olive seed lignin antioxidant capacity was calculated by evaluating free radical scavenging effect against DPPH free radical according to the earlier methods [6] with slight modifications. Antioxidant Activity (AA) (%) of olive seed lignin was calculated from the data of DPPH inhibition using UV-Vis spectra. The antioxidant activity (%) is expressed using Equation 1:

*Antioxidant Activity (AA)(%)=100 x (A*_*o*_*-A*_*s*_*)/A*_*o*_, (1)

where AA% is the antioxidant activity, *A*_*o*_ is the absorbance of the control and *A*_*s*_ is the absorbance of lignin sample. AA% is expressed in terms of percentage of inhibition. Figure 10d and Table 3 summarizes the antioxidant activity percent of the olive seed lignin before and after cryomilling (from 5 to 60 min). Clearly, the antioxidant activity of olive seed lignin enhances with longer cryomilling (due to generation of new radicals) and longer waiting time (due to migration of radicals to the surface). Noncryomilled lignin samples with the 2.37% of antioxidant activity from the start, reaches up to 40.07% at the end of 96 h of waiting in the DPPH solution, while 60 min cryomilled samples have 18.86% of antioxidant activity that reaches to 87.25% at the end of waiting duration. 

**Table 3:** Antioxidant activity (%) of the olive seed lignin samples cryomilled various times after different waiting durations in the DPPH solution.

Antioxidant activity (AA) (%)*					
Waiting duration (h)	Cryomilling duration (min)				
	0	5	10	30	60
0	2.37	4.30	8.34	21.13	18.86
6	7.87	14.29	21.94	36.62	45.95
24	18.83	24.06	30.60	46.62	64.78
48	28.93	36.85	47.10	61.74	74.12
72	40.95	45.80	50.27	63.51	80.99
96	40.04	46.46	55.69	68.24	87.25

*Note: AA is expressed in terms of percentage inhibition.

Our results show that the olive seed lignin antioxidant activity is about 15% more than that of Alu’datt et al. which various temperature, time, solvent and extraction method were tried and the highest antioxidant activity was recorded to be 73.67% [18]. 

## 3. Conclusion

In this paper, extraction and characterization of olive seed lignin were discussed, additionally quantification of mechanochemically formed radicals are evaluated. Initially, lignin is extracted from olive seed powders by acid extraction method. Isolated lignin was then mechanochemically grinded in cryo conditions and then determination of particle size was carried out by using Zeta Sizer. As expected, the size of lignin particles was reduced (initially 1 μm to about 450 nm in solution) by milling at the start, nevertheless further milling raised it (around 800 nm) due to aggregation in solution. Unchanging of the amorphous behaviour of lignin during cryomilling was confirmed by XRD. Formation of lignin mechanoradicals due to bond breakages during cryomilling were confirmed by FTIR-ATR and no further structural transformations were observed. ESR studies supported FTIR-ATR and confirmed β-O-4 bond breakage and the phenoxy radical formation (with a detection of g-value of 2.0025) on the cryomilled olive seed lignin. TGA and DSC analysis revealed that lignin starts to decompose slightly below 200 °C and two glass transitions (at 57.5 °C and 68.5 °C) were observed, indicating two different amorphous phases. After isolated lignin were acylated, ^1^H‑NMR and GPC were carried out; M_n_ and M_w_ were found to be around 5078 and 19,250; respectively with a PDI of 3.79. Quantitative analysis of radicals created during mechanochemical grinding was calculated using the decrease in the absorption intensity of the DPPH -radical scavenger- solution at its maximum wavelenght of by using UV-Vis. The amount of radicals per gram of noncryomilled lignin is calculated to be around 7.64 × 10^15^ while it is around to be 1.48 × 10^16^ for 60 min cryomilled lignin; concluding that about two times more radicals are created with 60 min cryomilling.

## 4. Experimental 

### 4.1. Materials

Olive seed (originated from Kocaeli, Turkey) was purchased via commercially available sources as crushed powder. Sulfuric Acid (initially 95%–97%, diluted to 72%), Acetic Acid (99.85%), trimethylacetylchloride, ACN and DPPH (2,2-diphenyl-1-picrylhydrazyl) bought from Sigma-Aldrich Corp. (St. Louis, MO, USA). Autoclave Reactor (PARR Instrument Company, Moline, IL, USA) was used in extraction processes of lignin from grinded olive seeds. Retsch Molecular Sieves and Retsch Cryomill Grinder (Retsch GmbH, Haan, Germany) with LN_2_ tank were used to grind and cryomill the samples. 467,000–205,000–72,450–29,510 and 10,210 g/mol PS standards (Varian Medical Systems Inc, Palo Alto, CA, USA) were used for calibration curve for GPC measurements.

### 4.2. Extraction of lignin via Klason method

In general, lignin is separated from wood-like natural sources either by removing non-lignin components (Klason, Willstatter, periodate or cuproxam lignin) or by removing lignin component (Brauns native lignin-BNL, cellulolytic enzyme lignin-CEL or milled wood lignin-MWL- in other name Bjorkman lignin) [39].

Depending on the Bjorkman lignin preparation method, the lignin content can be extracted easily if the biomass is milled [12]. Thus, we extracted lignin of olive seed starting from finely grinded olive seed powder. The lignin content of olive seed was determined in accordance with Tappi T222 om-88 [40]. In this method, lignin known as “Klason lignin or sulfuric acid lignin” is defined as the wood component insoluble in a 72% sulphuric acid solution. Olive seed powder was sieved out by using a 500 μm Retsch Molecular Test Sieves (Retsch GmbH) in order to comb out larger particles. Five grams of olive seed was mixed with 10 mL of 72% H_2_SO_4_ solution (Sigma-Aldrich Corp.). 72% H_2_SO_4_ solution was selected in order to enable the decomposition of any carbonhydrates, waxes, resins, oil etc. while avoiding the degradation of lignin itself. The ‘acid bomb’ technique was applied by keeping the mixture in an autoclave reactor at 150 °C for 45 min. The mixture was carefully diluted with water after the autoclave reactor was cooled to room temperature. Black solid was washed several times with large amount of water using suction filtration, pH value of 4–5 was confirmed and lignin sample was obtained as insoluble black residue after drying under vacuum at 30 °C. 

### 4.3. Grinding

Extracted lignin was first crushed using a mortar and pastel for a few minutes. In order to further reduce the particle size, a Retsch Cryomill instrument was used (Retsch GmbH). Low temperature is achieved by liquid nitrogen that circulates through the milling chamber; thus, efficient milling is applied without sticking on the walls of the grinding chamber. By default setting of the instrument, the cryomilling starts only when the cryo condition is reached. And the instrument is both equipped with the digital temperature control and the automatic shutdown for possible LN_2_ deficiency. Lignin was milled with 6 zirconia balls (each 10.06 mm diameter) as grinding medium in a zirconia grinding chamber (25 ml volume) at 30 Hz frequency (5-min precooling at 5 Hz was applied prior to each sample) for an indicated time at cryo conditions of –196 °C. Lignin samples of 0.250 g were cryomilled for 5, 10, 30 and 60 min.

### 4.4. Synthesis of acylated lignin

Derivatization is typically accomplished by acylation with slight modification from reports in literature [28]. Twenty milligrams of olive seed lignin was cryomilled for 60 min and dispersed in 20 mL of 1, 4‑dioxane and placed in round bottom flask. The mixture was stirred while 5 mL of acetic acid was added dropwise and mixed overnight at room temperature. 10 mL of trimethylacetylchloride was added and the mixture was refluxed for 3 days. Solution was cooled down to room temperature and acetic acid/excess trimethylacetylchloride were removed first by rotary evaporator and then by high vacuum drying at 40 °C overnight. Acylated lignin is recovered as brown-black solid powder.

### 4.5. Characterization of extracted lignin

Particle size distribution analyses of lignin particles extracted from olive seeds were done with Malvern Zeta Sizer instrument after about 5 mg of each sample was mixed with 5 mL of 1,4 dioxane. The technique allows the determination of the sizes of particles in a range of 5 nm to 50 µm by laser diffraction method. Each measurement was performed three times (each time 16 cycles were averaged by the instrument) and average values were collected. 

Number and weight average molecular weight (M_n_ and M_w_) of acylated lignin and polydispersity index (PDI) were estimated by gel permeation chromatography (GPC) (Agilent 1260 Infinity, 2 Agilent PL gel columns, Agilent Technologies, Inc., Santa Clara, CA, USA) in THF at 40 °C with a flow rate of 0.6 ml/min and an injection volume of 20 μL. A calibration curve was obtained by using polystyrene standards. 

The structural changes of the lignin extracted from olive seed before cryomill and after different cryomilling times were determined by using Bruker Alpha FTIR-ATR Spectrometer (Bruker Corp., Billerica, MA, USA). Spectral width of 4.000 cm‑1–400 cm^-1^, 32 scans at a resolution of 4 cm‑1. 

Cary 300 UV‑Vis spectrophotometer was used to track the absorbance change of the lignin-DPPH-ACN solutions. 0.250 mg of cryomilled lignin (for 5 to 60 min) were added into 50 μL 1.3 × 10‑1 M DPPH solutions prepared in ACN. Then the mixture was diluted to 5 mL (a stock DPPH-lignin solution). From the stock solution, 0.5 mL of DPPH‑lignin solution was taken and diluted to 5 mL with ACN. Following the same dilution process, a control sample containing DPPH without lignin was also prepared. The absorbance of the final solutions was followed via UV-Vis spectrophotometer after an indicated waiting time (the samples were kept in dark in PP tubes in order to avoid DPPH bleaching). In order to calculate the standard deviation, each measurement was repeated at least 3 times with new samples. From the same data of the absorption inhibition data of olive seed lignin samples in DPPH, antioxidant activity can be calculated similarly. Olive seed lignin antioxidant capacity was calculated by evaluating free radical scavenging effect against DPPH free radical according to the earlier methods [6] with slight modifications. Antioxidant Activity (AA) (%) of olive seed lignin was calculated from the data of DPPH inhibition using UV-Vis spectra. The DPPH radical scavenging activity or in another term, antioxidant activity (%) is expressed using Equation 1.

The XRD measurements were performed on an X’Pert PRO, PANalytical model X-ray diffractometer (Malvern Panalytical B.V., Almelo, Netherlands) with Cu Kα radiation. Forty milliamperes applied current and 45 kV accelerating voltage were used. The X-ray scanning was performed at diffraction angles of 2-Theta (from 5° to 80°, with increments of 0.02°).

Thermal stability of the lignin extracted from olive seed was investigated by using TGA Q500 from TA Instruments, Inc. (New Castle, DE, USA). In order to get rid of the moisture contribution, the dry sample was heated from ambient temperature to 100 °C and 10 min are given to completely remove it. The sample temperature was equilibrated back to room temperature and finally heated up to 900 °C with a heating rate of 20 °C/min and the weight loss during the final heating was recorded only. 

Thermal transition temperature analysis of the olive seed lignin was studied by using DSC Q2000 from TA Instruments, Inc. The sample was heated from room temperature to 200 °C with a heating rate of 20 °C/min and thermal behaviour over heating and cooling back was recorded. In order not to clear the thermal history, no preheating is applied. The sample is used as it was extracted from olive seeds. 

The surface topography of olive seed lignin was imaged with a FEI Quanta 200F model SEM with an accelerating voltage of 15 kV. 20 nm Au/Pd was sputtered on the samples prior to the SEM imaging.^1^H-NMR spectra were recorded on Bruker Biospin Avance 400 NMR spectrometer at 400 MHz in CDCl3. TMS was used as the internal reference.

Electron spin resonance (ESR) or electron paramagnetic resonance (EPR) spectroscopy analysis were performed for the as received olive seed powder, 60 min cryomilled and noncryomilled lignin extracted from the olive seed powder. Mechanochemically generated radicals were characterized at room temperature under nonsaturating conditions using Bruker ELEXYSY E580 model ESR spectrometer equipped with a high sensitivity cavity and operating at X-band frequencies (9 GHz). Intensities of the ESR signal were obtained by double integration of the corrected baseline using Bruker Win EPR software. Experimental conditions were set to: 12 dB, 1G, 0.3 mW microwave power, 0.25 mT modulation amplitude and 1024 points.

## References

[ref1] (2018). Olive oil value-chain dynamics: the Turkish olive oil industry case. Acta Horticulturae.

[ref2] (2008). Olive stone an attractive source of bioactive and valuable compounds. Bioresource Technology.

[ref3] (1987). Olive stones as a source of fermentable sugars. Biomass.

[ref4] (2007). Radical scavenging capacity of lignin and its effect on processing stabilization of virgin and recycled polypropylene. Journal of Applied Polymer Science.

[ref5] (1998). Free radical-scavenging properties of lignin. Nutrition and Cancer.

[ref6] (2012). Investigation of the effects of different organosolv pulping methods on antioxidant capacity and extraction efficiency of lignin. Food Chemistry.

[ref7] (2017). Natural antioxidants as stabilizers for polymers. Polymer Degradation and Stability.

[ref8] (2015). developments in polymers derived from industrial lignin. Journal of Applied Polymer Science.

[ref9] (2002). Recent industrial applications of lignin: a sustainable alternative to nonrenewable materials. Journal of Polymers and the Environment.

[ref10] (2010). Study of the antioxidant capacity of miscanthus sinensis lignins. Process Biochemistry.

[ref11] (2010). Effects of process severity on the chemical structure of miscanthus ethanol organosolv lignin. Polymer Degradation and Stability.

[ref12] (1956). A. Studies on finely divided wood. Part 1. Extraction of lignin with neutral solvents. Svensk Pepperstein.

[ref13] (1992). Methods in Lignin Chemistry.

[ref14] Isolation of lignin.

[ref15] (2000). The antioxidant/anticancer potential of phenolic compounds isolated from olive oil. European Journal of Cancer.

[ref16] (2008). Radical scavenging-linked antioxidant activity of ethanolic extracts of diverse types of extra virgin olive oils. Journal of Food Science.

[ref17] (2001). Antioxidants and total peroxyl radical-trapping ability of olive and seed oils. Journal of Agricultural and Food Chemistry.

[ref18] (2011). Optimisation and characterisation of various extraction conditions of phenolic compounds and antioxidant activity in olive seeds. Natural Product Research.

[ref19] (1967). Formation of solid free radicals by mechanical action. Nature.

[ref20] (1992). A generation mechanism of triboelectricity due to the reaction of mechanoradicals with mechanoions which are produced by mechanical fracture of solid polymer. Colloid and Polymer Science.

[ref21] (2013). Mechanochemical degradation of lignin and wood by solvent-free grinding in a reactive medium. Green Chemistry.

[ref22] (2020). A sustainable preparation of catalytically active and antibacterial cellulose metal nanocomposites via ball milling of cellulose. Green Chemistry.

[ref23] (2020). Why does wood not get contact charged? lignin as an antistatic additive for common polymers. Chemistry of Materials.

[ref24] (1956). : a theory based on free radical and radiation chemistry. Journal of Gerontology.

[ref25] (2018). Preparation and characterization of starch nanocrystals combining ball milling with acid hydrolysis. Carbohydrate Polymers.

[ref26] (2011). Evidence of micro- and nanoscaled particles during starch nanocrystals preparation and their isolation. Biomacromolecules.

[ref27] (2014). X-Ray diffraction analysis of kraft lignins and lignin-derived carbon nanofibers. Journal of Nanotechnology in Engineering and Medicine.

[ref28] (2012). Accurate and reproducible determination of lignin molar mass by acetobromination. Journal of Agricultural Food Chemistry.

[ref29] (2000). Piassava fibers (Attalea funifera): NMR spectroscopy of their lignin. Journal of the Brazilian Chemical Society.

[ref30] (2016). Nitrobenzene oxidation for isolation of value added products from industrial waste lignin. Journal of Chemical, Biological and Physical Sciences.

[ref31] (2018). ESR estimation of lignin structure of japanese cedar pulverized using a vibration mill with ring media. Journal of the Japan Institute of Energy.

[ref32] (1960). Electron paramagnetic resonance studies of stable free radicals in lignins and humic acids. Nature.

[ref33] (2004). Free radicals in the photolysis and radiolysis of polymers: IV. Radicals in γ- and UV-irradiated wood and lignin. High Energy Chemistry.

[ref34] (2000). Electron spin resonance studies on paramagnetic species produced by milling of woods: detection of phenoxy radicals and Mn2+ ions. Mokuzai Gakkaishi.

[ref35] (2016). Title characterization of free radicals by electron spin resonance spectroscopy in biochars from pyrolysis at high heating rates and at high temperatures. Biomass and Bioenergy.

[ref36] (1978). Electron spin resonance spectrometric study of free radicals in coals. Analytical Chemistry.

[ref37] (1958). Antioxidant Determinations by the Use of a Stable Free Radical. Nature.

[ref38] (1995). Use of a free radical method to evaluate antioxidant activity. LWT - Food Science and Technology.

[ref39] (1962). Chemistry of lignin.

[ref40] T222 om-88. TAPPI Test Method T222 Om-88, Acid-Insoluble Lignin in Wood and Pulp. In: Tappi Test Methods. Atlanta, GA: Technical Association of the Pulp and Paper Industry..

